# A real opportunity to modify cardiovascular risk through primary care and prevention: A pilot study

**DOI:** 10.3389/fpubh.2022.1009246

**Published:** 2023-01-10

**Authors:** Alberto Lontano, Eleonora Marziali, Caterina Galletti, Eduardo Mazza, Stefano Gambioli, Valerio Galasso, Alessandro Mingarelli, Floriana D'Ambrosio, Andrea Tamburrano, Massimo Paolini, Antonio Bande, Gianfranco Damiani, Chiara de Waure, Patrizia Laurenti

**Affiliations:** ^1^Università Cattolica del Sacro Cuore, Rome, Italy; ^2^Azienda Ospedaliera San Camillo Forlanini, Rome, Italy; ^3^DiagnostiCare ONLUS, Rome, Italy; ^4^Scuola di Specializzazione in Psicologia della Salute–Università degli Studi di Roma “La Sapienza”, Rome, Italy; ^5^Health Management IRCCS San Raffaele Roma, Rome, Italy; ^6^Fondazione Policlinico Universitario Agostino Gemelli IRCCS, Rome, Italy; ^7^Department of Medicine and Surgery, University of Perugia, Perugia, Italy

**Keywords:** cardiovascular disease, cardiovascular risk score, risk reduction, primary care, primary prevention, health promotion

## Abstract

Cardiovascular diseases (CVDs) represent a major threat to health and primary prevention outstands as the most effective instrument to face this issue, addressing multiple risk factors at a time and influencing behavioral patterns. Community nurses have been involved in many interdisciplinary prevention activities, resulting in effective control of CV risk factors. We conducted a pilot study aiming at describing the impact on the CV risk profile of an 18-month interdisciplinary intervention on lifestyle habits. From September 2018 to May 2020, four general practitioners (GPs) working in the Roman neighborhood of Torresina recruited patients having a cardiovascular risk score (CRS) equal to or higher than 3% and lower than 20%; those patients were included in a nutritional, physical, and psychological counseling program. Assessments of patients' health status were led at baseline, 6, 12, and 18 months by a nutritionist, a physiotherapist, a psychologist, their GPs, and a community nurse. The CRS was estimated at every examination, based on the Italian Progetto Cuore algorithm. A total of 76 patients were included (mean age of 54.6 years; 33 men and 43 women). Mean CRS showed a significant reduction between baseline and 12 months (from 4.9 to 3.8); both total cholesterol and systolic blood pressure (SBP) significantly decreased at 6 months of follow-up (respectively, from 211.1 to 192 and from 133.1 to 123.1). Nonetheless, the reduction was later maintained only for SBP. However, during the last 6 months of the intervention, the COVID-19 pandemic broke out, thus, it is not possible to know how much the results achieved at 18 months were influenced by the restrictive measures introduced by the Italian government. When stratifying according to the presence of hypertension/diabetes and physical activity, no differences in the CRS could be highlighted between the two groups. Our pilot study proved that an interdisciplinary counseling intervention program can improve CV risk profile and could be further spread to people that, according to their CRS, would benefit more from changes in lifestyles.

## 1. Introduction

Cardiovascular diseases (CVDs) are a group of disorders affecting the heart and blood vessels (1), which represent the leading cause of death, premature death, and reduced quality of life globally: in 2019, they were responsible for an estimated 17.9 million deaths, among which 3.9 million in Europe alone (1). It has been estimated that one person dies every 36 s in the United States due to CVDs (2), with ischemic heart disease and stroke being the most frequent conditions (3). Moreover, not only do CVDs determine a significant and avoidable number of human lives lost, but they also outline a substantial economic burden, which cost the EU €210 billion a year in 2017, considering direct healthcare costs, productivity losses, and informal care of people with CVD (2).

“Progetto Cuore” is an Italian epidemiological project financed by the Italian Ministry of Health and aimed at recording CV events and the distribution of CV risk factors, in order to identify strategies to lower CV risk and consequent morbidity and mortality (4). In this regard, influencing behavioral patterns through primordial and primary prevention has been appointed as a decisive strategy (5, 6). As described in a systematic review by Sisti et al. addressing more than a single risk factor at a time can be more effective in CV risk reduction, particularly in individuals at risk: interventions on diet and physical activity, together with quitting tobacco use, are the ones that impact the most (7, 8).

Primary care is a suitable setting to identify patients who might be at risk of developing CVDs (9). Risk stratification is intended to target the intensity of the therapeutic or preventive measures to the severity of the patient's risk (10) and is complementary to the preventive population approach, which deals with population-wide strategies independently of individual risk (11). General practitioners (GPs) can assess CV risk by using validated risk scores (9, 10), such as SCORE (Europe) (12), Framingham (13), and ASCVD (US) (14), based on different combinations of modifiable risk factors that are also targets for prevention (15–19). By means of risk stratification, cardiovascular risk scores (CRS) allow one to plan lifestyle interventions, for instance, physical activity, diet, and smoking interventions (15). In several cases, interdisciplinary prevention activities have been coordinated by community nurses, creating bridges between primary care and hospitals (20): virtuous experiences have proved that primary prevention can be led outside of specialist centers and it can result in significant improvements in lifestyles and more effective control of risk factors (20).

This pilot study aims at describing the impact of a 12-month prevention and promotion, interdisciplinary program on lifestyle habits, and CV risk assessed through the CRS developed by “Progetto Cuore.” The intervention was led in a primary care setting in the Roman neighborhood of Torresina.

## 2. Materials and methods

### 2.1. Study design and population

“Cardiovascular Risk Prevention in the Neighborhood Torresina” is an 18-month interdisciplinary pilot pre-post observational study conducted among healthy individuals in the Roman neighborhood of Torresina [~5,000 inhabitants (21)] from December 2018 to May 2020.

The sample was recruited in September 2018 among the patients of the four GPs working in the Torresina neighborhood. The study envisaged the organization of an information campaign in order to sponsor the scientific initiative through the websites of the Torresina neighborhood and of the DiagnostiCare ONLUS Association, involved in the project. Posters about the project and the selection criteria of the participants were hung in the medical practices involved and in the most frequented strategic points of the neighborhood; moreover, flyers with the same information were placed in letterboxes in the neighborhood. Participants were recruited among all men and women aged between 35 and 69 years of age who presented at the GP practices in September 2018: among them, those who consented to enroll voluntarily and fulfilled the inclusion criteria took part in the study. The fact that a commitment was required for a medium to a long time further contributed to the self-selection of participants.

### 2.2. Parameters sampling

To estimate CRS, standardized methods were used to assess parameters according to “Progetto Cuore.” To standardize the measurements, blood samples were taken in a laboratory specifically identified for the study.

#### 2.2.1. Fasting blood glucose

To determine fasting blood glucose, a blood venous withdrawal was carried out after at least a 12-h fasting. To be diagnosed with diabetes, the patient must have a fasting blood sugar level equal to or >126 mg/dL in 2 or more consecutive tests within a week or be treated with oral hypoglycemic drugs or insulin.

#### 2.2.2. Blood pressure

Systolic blood pressure (SBP) was measured according to 2017 ACC/AHA/AAPA/ABC/ACPM/AGS/APhA/ASH/ASPC/NMA/PCNA guidelines (22). It was determined two times within few minutes. The blood pressure value was calculated as the mean of the two readings.

#### 2.2.3. Total, HDL, and LDL cholesterol and triglycerides

To determine total, high-density lipoprotein (HDL), and low-density lipoprotein (LDL) cholesterol and triglycerides, a blood venous withdrawal was carried out after at least a 12-h fasting.

#### 2.2.4. Smoking habit

A smoker was defined as a person who smokes tobacco regularly every day (even only one cigarette) or has quitted for <12 months.

#### 2.2.5. Physical activity

Physical activity was defined as having performed at least 150 min of moderate-intensity aerobic physical exercise, at least 75 min of vigorous-intensity aerobic physical exercise, or an equivalent combination of moderate- and vigorous-intensity activity throughout the week.

#### 2.2.6. Alcohol consumption

Moderate drinkers were defined as those limiting their intake to 2 drinks or less per day for men and 1 drink or less per day for women when alcohol was consumed (23). Heavy drinkers were defined as men consuming more than 4 drinks on any day or more than 14 drinks per week, or women consuming more than 3 drinks on any day or more than 7 drinks per week (23).

#### 2.2.7. Cardiovascular risk score determination

Global absolute CV risk is a parameter that defines the probability (in %) of the occurrence of a major CV event in the following 10 years in the adult population aged between 35 and 69 years. The software “Cuore.exe” (24), developed by the Italian National Institute of Health (ISS—Istituto Superiore di Sanità), was employed to determine the individual CRS, which is the best estimate of the global absolute CV risk. The software refers to 8 parameters, namely, age, gender, diabetes, smoking habit, SBP, total and HDL cholesterol, and antihypertensive drugs.

### 2.3. Data sources and management

All measurements, including the determination of the individual CRS, were carried out at baseline and at 6, 12, and 18 months by GPs and a community nurse.

Total blood glucose and cholesterol were assessed through laboratory testing. With respect to blood glucose, patients were not submitted to laboratory testing at baseline as GPs were already aware of their patient's status with respect to diabetes, thus, being allowed to gather the relevant information on the Cuore.exe software. Data concerning personal patient information (e.g., age, gender, educational level, profession, marital status, and nationality) and other relevant characteristics and prescriptions (e.g., smoking habits, diet, physical activity, and blood pressure) were collected by both GPs and community nurses. The 2016 “PASSI” questionnaire was used to collect information on lifestyles (25) while the Health Locus of Control (HLC) (26) and the Multidimensional Scale of Perceived Social Support (MSPSS) (27) were used to monitor behavioral changes. Participation in planned activities on lifestyle interventions was recorded by means of weekly diaries. All the information collected was organized in an Excel dataset, managed by the GPs: data were anonymized and identified through a univocal code.

The dataset was then handed over to the Institute of Public Health-Section of Hygiene of the Catholica University of the Sacred Heart of Rome for statistical analysis.

### 2.4. Health prevention and promotion intervention

The program was composed of three main interventions led by an interdisciplinary team at baseline, 6, and 12 months. Follow-up examinations were carried out at 6, 12, and 18 months.

#### 2.4.1. Intervention 1: Nutritional counseling

The nutritional intervention aimed to convey basic nutrition information, focusing on the correct Mediterranean dietary habits to reduce the risk of CVD. At first, through simple general questions, the level of awareness of the sample population regarding nutrition was measured. Then, the nutritionist explained the different classes of macronutrients and focused on foods that may contribute to increasing or decreasing the risk of CVD. The main topics addressed were body hydration, salt consumption, consumption of saturated and polyunsaturated fatty acids, fruits and vegetables, and alcoholic and soft drinks. Finally, advice and guidance were given on how to shop for groceries, teaching how to read food labels to better evaluate the nutritional information of packaged products. In addition, the nutritionist evaluated weekly nutritional diaries completed by the patients in order to assess and correct the most frequent mistakes.

#### 2.4.2. Intervention 2: Physical activity counseling

Relying on 2010 WHO “Global recommendations on Physical Activity for Health,” a physiotherapist and a motivational coach helped patients to develop an active lifestyle and organized group training activities.

In particular, training meetings were organized to make participants aware of their individual habits and to give advice and information on how to start and develop a training program based on walking.

Furthermore, a motivational coach set meetings to conduct outdoor group workouts and activities in the Torresina neighborhood area. These outdoor meetings were aimed at consolidating the group, creating an interrelationship among those involved, and sharing the benefits of doing physical activity together. This part of the program was carried out under the supervision of the physical therapist who provided professional support for the proper conduct of the practical activities.

#### 2.4.3. Intervention 3: Psychological counseling

The psychological intervention was based on the transtheoretical model of health behavior change (28) and aimed to promote the change of unhealthy behaviors toward the adoption of better self-care.

Experiential group meetings aimed at exploring individual expectations and promoting behavioral change were organized. In addition, the psychologist evaluated the diaries completed by the patients in order to develop corrective suggestions for the most frequent and overall errors noted.

### 2.5. Statistical analysis

Data were described through means and standard deviation (SD) and absolute and relative frequencies as appropriate. The comparison of values of laboratory, anthropometric, and pressure parameters, and CRS at baseline, 6, 12, and 18 months was performed through repeated measures ANOVA with *post-hoc* Bonferroni multiple comparison test. The sphericity assumption has been evaluated with Mauchly's test; in the case of violation, corrections have been adopted. Either the Greenhouse-Geisser or the Huynh–Feldt correction was used according to the epsilon estimate. Interaction terms were not included in the model as only one group of patients was assessed. The repeated measures ANOVA relied on the central limit theorem. The Friedman test was used to address differences in the proportion of smokers over time. All the analyses considered only patients having complete data throughout the different points in time and were also stratified according to the presence of diabetes or/and hypertension at baseline, considering that these two diseases are included in the calculation of the CRS but are not fully and timely modifiable. Another stratified analysis was performed with regard to physical activity. People were classified as physical activity if they self-reported in answering the PASSI questionnaire to make intense physical activity for more than 1 day a week or moderate physical activity for at least 5 days a week. The significance level was set at 0.05 and the SPSS software (version 22) was used for the analysis.

## 3. Results

The study population included 76 individuals, 33 (43.4%) men and 43 (56.6%) women. The mean age was 54.6 (±7.6) years and 16 individuals held a bachelor's or master's degree (21.1%), 36 held a high school diploma (47.4%), and 24 held a middle school diploma (31.6%). Among 74 respondents to the PASSI questionnaire, 61 declared to work (82.4%). Participants' health status and risk factors are shown in Table 1. The results of the repeated measurement analysis are shown in Table 2.

**Table 1:** Participants' health status and risk factors (N responders).

**Variable**	***N* (%) or mean (±SD)**
*Intense physical activity in the last 30 days* (29) Mean days of intense physical activity per week (21)	23 (31.5%) 2.8 (±1.7)
*Moderate physical activity in the last 30 days* (30) Mean days of intense physical activity per week (31)	51 (68.9%) 3.7 (±2.2)
*Smoking [76]*	
Ex-smokers for more than 1 year	18 (23.7%)
Current smokers	27 (35.5%)
Mean number of cigarettes smoked per day (27)	11.8 (±7.2)
*Alcohol*	
Assumption of at least 1 alcohol unit in a day in the last 30 days (32)	55 (90.1%)
Mean number of days with assumption of at least 1 alcohol unit in the last 30 days (33)	12.1 (±11.7)
Mean number of alcohol unit per day (33)	1.5 (±0.8)
*Receiving treatment for hypertension* [76]	16 (21.1%)
*Receiving treatment for hypercholesterolemia* (29)	8 (11.0%)
*Diagnosis of diabetes* (30)	4 (5.4%)
*Diagnosis of heart failure* (30)	0 (0%)
*Diagnosis of asthma* (30)	11 (14.9%)
*Diagnosis of COPD/emphysema/respiratory failure* (30)	5 (6.8%)
*Diagnosis of ischemic cerebrovascular diseases* (30)	1 (1.4%)
*Diagnosis of ischemic heart diseases* (30)	1 (1.4%)
*Diagnosis of other cardiovascular diseases* (30)	5 (6.8%)
*Diagnosis of cancer* (30)	1 (1.4%)
*Diagnosis of chronic hepatic diseases* (30)	1 (1.4%)
*Diagnosis of arthritis/arthrosis* (30)	16 (21.6%)

**Table 2:** Results of repeated measures ANOVA.

**Variable**	**Baseline mean (±SD)**	**6 months** **mean (±SD)**	**12 months** **mean (±SD)**	**18 months** **mean (±SD)**	***p***
Weight (kg) (32)	76.1 (±14.4)	75.0 (±4.7)	75.7 (±15.4)	75.5 (±15.2)	0.08
BMI (kg/m^2^) (32)	27.3 (±4.8)	26.8 ( 4.8)	27.1 (±5.1)	27.1 (±5.2)	0.07
Waist circumference (cm) (32)	90.4 (±12.3)	88.3 (±12.8)	90.4 (±13.5)	88.7 (±13.3)	**<0.001**
Glycemia (mg/dL) (34)	-	95.5 (±15.8)	100.4 (±15.3)	97.6 (±14.2)	0.06^∧^
Total cholesterol (mg/dL) (34)	211.1 (±32.8)	192.0 (±29.9)	202.5 (±37.8)	215.0 (±41.2)	**<0.001^∧^**
HDL cholesterol (mg/dL) (34)	56.6 (±14.2)	49.9 (±10.7)	57.4 (±13.7)	58.8 (±14.2)	**<0.001**
LDL cholesterol (mg/dL) (33)	136.1 (±27.8)	123.3 (±30.1)	128.4 (±31.9)	134.0 (±35.3)	**<0.01**
Triglycerides (mg/dL) (35)	101.1 (±54.9)	96.4 (±44.2)	92.7 (±41.3)	118.0 (±62.8)	**<0.01^∧^**
Systolic blood pressure (mmHg) (34)	133.1 (±17.1)	123.1 (±13.0)	122.8 (±13.0)	119.3 (±13.8)	**<0.001**
Diastolic blood pressure (mmHg) (34)	85.3 (±16.4)	77.5 (±7.5)	76.2 (±8.3)	72.9 (±8.1)	**<0.001^$^**
Smokers (N %) (32)	18 (±29.5)	17 (±27.9)	14 (±23.0)	16 (±26.2)	**0.04**
Cardiovascular risk score (34)	4.9 (±6.3)	3.7 (±3.2)	3.8 (±4.0)	4.0 (±4.0)	**0.02^$^**

$ Greenhouse-Geisser.

∧ Huynh–Feldt.

N.B. The value of glycemia at baseline is missing.

Statistically significant values are reported in bold.

The mean CRS was 4.9 (±6.3) at baseline and showed a significant reduction in time. The relative decrease from baseline to 18 months (calculated as the ratio of the difference between the two values and the baseline value) was 18.4%. The *post-hoc* multiple comparison test revealed that a significant difference in CRS has established between baseline and 12 months. Considering modifiable variables included in the calculation of the CRS, namely, smoking, SBP, total cholesterol, and HDL cholesterol, it emerged that total cholesterol and SBP significantly decreased at 6 months of follow-up. Nonetheless, the reduction has been maintained in the remaining points in time only for SBP. Also, the prevalence of smokers slightly decreased, in particular, at 12 months.

The analysis stratified according to the presence of diabetes and/or hypertension showed that the CRS did not significantly change in the two groups considered separately (Table 3). This could be due to the increase in glycemia that was observed in people without diabetes and/or hypertension, which could have countered the decrease in total cholesterol and systolic pressure. On the other hand, in people with diabetes/hypertension, results should be considered with caution because of the very low sample size. Actually, a 9% relative decrease in mean CRS was observed from baseline to 18 months in people without diabetes and/or hypertension as compared to 26.7% in people with diabetes and/or hypertension.

**Table 3:** Results of repeated measures ANOVA, stratified according to the presence of diabetes and/or hypertension.

	**Variable**	**Baseline** **mean (±SD)**	**6 months mean (±SD)**	**12 months** **mean (±SD)**	**18 months mean (±SD)**	***P***
No diabetic and/or hypertension patients	Weight (kg) (36)	74.2 (±12.8)	73.4 (±13.7)	73.7 (±14.3)	73.5 (±13.8)	0.27^∧^
	BMI (kg/m^2^)(36)	26.7 (±4.2)	26.4 (±4.6)	26.5 (±4.8)	26.4 (±4.9)	0.26^$^
	Waist circumference (cm) (37)	88.7 (±12.3)	86.2 (±11.6)	88.2 (±12.9)	86.3 (±12.6)	**<0.001**
	Glycemia (mg/dL) (36)	-	92.9 (±10.1)	96.9 (±10.1)	95.0 (±12.0)	0.03^∧^
	Total cholesterol (mg/dL) (36)	215.5 (±30.5)	196.9 (±31.2)	208.0 (±33.9)	221.9 (±39.1)	**<0.001^∧^**
	HDL cholesterol (mg/dL) (36)	57.4 (±15.2)	50.0 (±11.1)	57.1 (±13.6)	60.3 (±14.6)	**<0.001**
	LDL cholesterol (mg/dL) (38)	140.0 (±26.5)	127.2 (±30.6)	131.4 (±30.9)	136.8 (±35.8)	**0.02**
	Triglycerides (mg/dL) (37)	92.6 (±36.2)	90.6 (±33.7)	95.8 (±43.3)	116.6 (±58.3)	**<0.01^∧^**
	Systolic blood pressure (mmHg) (36)	129.5 (±15.9)	121.9 (±13.3)	122.1 (±13.6)	118.0 (±12.7)	**<0.001**
	Diastolic blood pressure (mmHg) (36)	83.0 (±15.7)	77.3 (±7.3)	75.6 (±8.5)	71.9 (±7.8)	**<0.001^$^**
	Smokers (N%) (37)	14 (±29.8)	13 (±27.7)	12 (±25.5)	13 (±27.7)	0.26
	Cardiovascular risk score (36)	3.2 (±3.0)	2.8 (±2.5)	2.8 (±2.3)	2.9 (±2.7)	0.12^$^
Diabetic and/or hypertension patients	Weight (kg) (13)	83.1 (±18.0)	80.8 (±17.3)	82.8 (±17.8)	82.9 (±18.1)	0.25
	BMI (kg/m^2^) (13)	29.5 (±5.7)	28.7 (±5.5)	29.3 (±5.6)	29.4 (±5.7)	0.25
	Waist circumference (cm) (14)	96.0 (±11.0)	95.3 (±14.5)	97.6 (±13.4)	96.4 (±13.1)	0.35
	Glycemia (mg/dL) (14)	-	104.6 (±26.3)	112.4 (±23.0)	106.4 (±17.7)	0.50^$^
	Total cholesterol (mg/dL) (14)	195.8 (±37.0)	175.4 (±17.2)	183.6 (±45.3)	191.3 (±40.7)	0.12
	HDL cholesterol (mg/dL) (14)	53.9 (±10.3)	49.7 (±9.7)	58.5 (±14.4)	53.7 (±11.9)	**<0.01**
	LDL cholesterol (mg/dL) (10)	118.6 (±28.4)	105.7 (±20.9)	114.9 (±34.2)	121.4 (±32.1)	0.18
	Triglycerides (mg/dL) (13)	132.1 (±92.0)	117.3 (±68.2)	81.4 (±31.8)	122.8 (±79.4)	0.07^$^
	Systolic blood pressure (mmHg) (14)	145.6 (±15.5)	127.2 (±11.4)	124.9 (±10.8)	123.5 (±16.7)	**<0.001**
	Diastolic blood pressure (mmHg) (14)	93.1 (±17.0)	78.5 (±8.6)	78.4 (±7.2)	76.5 (±8.5)	**<0.01^$^**
	Smokers (*N*%) (14)	4 (±28.6)	4 (±28.6)	2 (1 ± 4.3)	3 (±21.4)	0.19
	Cardiovascular risk score (14)	10.5 (±10.6)	6.7 (±3.8)	7.0 (±6.4)	7.7 (±5.5)	0.10^$^

$ Greenhouse-Geisser.

∧ Huynh–Feldt.

N.B. The value of glycemia at baseline is missing.

Statistically significant values are reported in bold.

The analysis stratified according to physical activity showed that the CRS did not significantly change in the two groups considered separately (Table 4). The trend in total and HDL cholesterol as well as systolic pressure were confirmed with respect to the overall study population. A 10.8% relative decrease in mean CRS was observed from baseline to 18 months in physically active people as compared to 22.4% in physically inactive people. Interestingly, physically active people also showed a significant decrease in weight and BMI at 6 months.

**Table 4:** Results of repeated measures ANOVA, stratified according to the physical activity status.

	**Variable**	**Baseline** **mean (±SD)**	**6 months mean (±SD)**	**12 months** **mean (±SD)**	**18 months mean (±SD)**	***P***
Physically active	Weight (kg) (28)	75.7 (±15.1)	73.5 (±14.6)	74.5 (±15.5)	74.8 (±15.2)	**0.01**
	BMI (kg/m^2^) (28)	27.3 (±5.1)	26.5 (±5.1)	26.9 (±5.4)	27.1 (±5.5)	**0.01**
	Waist circumference (cm) (27)	89.9 (±12.8)	87.6 (±12.8)	88.9 (±12.8)	88.0 (±13.2)	**0.04^∧^**
	Glycemia (mg/dL) (28)	-	96.3 (±11.1)	101.1 (±11.3)	99.4 (±14.9)	**0.04**
	Total cholesterol (mg/dL) (28)	220.3 (±28.7)	192.0 (±28.9)	210.1 (±33.9)	218.1 (±28.6)	**<0.001**
	HDL cholesterol (mg/dL) (28)	56.9 (±11.5)	49.1 (±8.2)	58.5 (±12.1)	60.1 (±12.1)	**<0.001**
	LDL cholesterol (mg/dL) (25)	142.3 (±25.2)	121.3 (±29.4)	132.5 (±31.4)	135.6 (±27.8)	**<0.001**
	Triglycerides (mg/dL) (27)	106.4 (±57.9)	102.2 (±43.4)	95.3 (±38.5)	112.9 (±54.1)	0.38
	Systolic blood pressure (mmHg) (28)	129.0 (±16.2)	121.4 (±11.3)	121.1 (±10.2)	119.5 (±11.2)	**0.001**
	Diastolic blood pressure (mmHg) (28)	81.9 (±17.4)	77.6 (±7.4)	74.9 (±7.2)	74.1 (±7.7)	**0.03^$^**
	Smokers (N%) (27)	6 (±22.2)	6 (±22.2)	6 (±22.2)	6 (±22.2)	N.C.
	Cardiovascular risk score (28)	3.7 (±3.0)	3.1 (±2.4)	3.2 (±2.5)	3.3 (±2.8)	0.12^$^
Physically inactive	Weight (39)	77.3 (±14.6)	76.9 (±15.8)	77.1 (±16.5)	76.6 (±16.3)	0.64
	BMI (39)	27.2 (±4.4)	27.1 (±4.8)	27.1 (±5.0)	26.9 (±5.0)	0.62
	Waist circumference (40)	90.8 (±12.9)	88.9 (±13.7)	91.2 (±15.0)	88.9 (±14.3)	**0.001**
	Glycemia (mg/dL) (40)	-	95.8 (±20.0)	100.1 (±19.3)	96.0 (±14.5)	0.41^$^
	Total cholesterol (mg/dL) (40)	202.9 (±32.9)	189.1 (±31.3)	191.4 (±36.8)	209.3 (±49.1)	**0.01^$^**
	HDL cholesterol (mg/dL) (40)	55.6 (±16.6)	48.7 (±11.5)	54.6 (±14.2)	56.9 (±15.6)	**<0.001**
	LDL cholesterol (mg/dL) (27)	129.2 (±29.0)	123.9 (±32.2)	120.7 (±29.5)	128.6 (±40.5)	0.36
	Triglycerides (mg/dL) (39)	98.2 (±55.5)	96.2 (±45.9)	91.9 (±45.4)	127.1 (±72.4)	**<0.001**
	Systolic blood pressure (mmHg) (40)	136.1 (±17.7)	124.9 (±15.2)	122.6 (±15.2)	120.0 (±16.3)	**<0.001**
	Diastolic blood pressure (mmHg) (40)	88.3 (±16.1)	77.2 (±8.1)	77.1 (±9.1)	71.6 (±8.5)	**<0.001^$^**
	Smokers (N%)(40)	10 (±33.3)	10 (±33.3)	7 (±24.3)	9 (±30.0)	0.07
	Cardiovascular risk score (40)	5.8 (±8.3)	4.3 (±3.8)	4.0 (±4.7)	4.5 (±4.5)	0.11^$^

$ Greenhouse-Geisser.

∧ Huynh–Feldt.

N.B. The value of glycemia at baseline is missing.

NC, not calculable.

Statistically significant values are reported in bold.

## 4. Discussion

According to the “2019 ACC/AHA Guideline on the Primary Prevention of Cardiovascular Disease,” the most important way to prevent CVDs is to promote a healthy lifestyle throughout life (39). Intervening on modifiable risk factors to reduce total CV risk is possible through regular physical activity (40), maintenance of adequate weight, proper nutrition (41, 42), and abstention from tobacco smoking (42–44). Such lifestyle modifications in many cases require changes in behavior, and it is, therefore, important that health professionals employ effective behavioral strategies for the overall lifestyle management of their patients (45). Addressing this issue requires building a coalition of health disciplines, including physicians, nurses, health professionals, and psychologists allied to help prevent and control non-communicable diseases (45, 46). To be effective, this type of approach must not stop at multidisciplinarity but aspire to interdisciplinarity, in which healthcare professionals with different backgrounds interpenetrate their knowledge at different levels to provide care that has the patient at its center (46, 47).

In our study, we represent an interdisciplinary community approach model of primary prevention through tailored educational interventions resulting in a significant reduction of CRS. This reduction, observed between baseline and 6–12 months, was due to a similar trend observed in some parameters that are used to calculate the CRS. In a systematic review, Pennant et al. identified 36 observational studies on community programs for the prevention of CVDs: these programs combined media, screening, and environmental changes. Although not *via* meta-analysis, they described an average net reduction of 9.08% in 10-year CVD risk (47); however, this value is lower than the one outlined in our study (22%). Stewart et al. (44) and Patnode et al. (48) showed that even a small reduction in SBP is associated with a significant reduction in CVD risk; to this proposition, Lewington et al. (49) showed that CVD risk doubles for every 20 mmHg increase in SBP and 10 mmHg increase in DBP in all age and ethnic groups. In a review of the literature, Sisti et al. reported a significant reduction in SBP and DBP of −5.20 and −4.53 mmHg, respectively, as a result of a lifestyle intervention at 6 months. Again, the reduction was also significant at 12 months (−3.34 and −2.98 mmHg, respectively) (7). These results are similar to ours; nevertheless, the change in pressure values was higher in our study both at 6 and 12 months. Patients in our study also showed better SBP reduction than those in a study by Lönnenberg et al. when compared after 12 months of follow-up (50). SBP and DBP are also impacted by diet and exercise as illustrated by Patnode et al. (48) in a primary care intervention, showing a decrease of approximately 1.3 and 0.5 mmHg in SBP and DBP at 6–12 months of follow-up compared with the usual care. Moreover, Sisti et al. assessed a significant reduction in total cholesterol (−0.26 and −0.21 mmol/L at 6 and 12 months, respectively) and triglycerides (−0.19 and −0.09 mmol/L at 6 and 12 months, respectively) (7): although this matches our results for cholesterol, we were not able to show significant changes in relation to triglycerides (7). Patnode et al. also outlined that dietary and physical activity counseling decreases LDL cholesterol level by approximately 2.6 mg/dl and total cholesterol level by approximately 2.8 mg/dl at 6–12 months of follow-up compared with usual care. In particular, they demonstrated that high-intensity interventions result in a greater reduction of LDL and total cholesterol, with no evidence of an effect on HDL cholesterol or triglyceride level (48). Quitting smoking is a relevant cost-effective intervention in primary prevention (51). As a consequence of our interdisciplinary program, we report an initial reduction in the number of smokers, which aligns with the net reduction in smoking prevalence of 1.7% described by Pennant et al. in their systematic review (47).

However, despite an initial improvement in values observed during the first 12 months of intervention, values at 18 months did not show significant differences with respect to baseline. In the last phase of our study, an external event such as the COVID-19 pandemic intervened to alter the effects of our program in the most critical phase, i.e., that of self-maintenance of patients of the results achieved. In light of the emergency situation, the Italian authorities have planned a series of restrictive measures, including a national lockdown in the period of March–April 2020, travel bans, the institution of work from home, and closures of shops and services (52): this resulted in an increase in sedentary behavior and increased CV risk by acting on modifiable risk factors (38, 53). In fact, a sedentary lifestyle is associated with increased oxidative stress and metabolic dysfunction that can result in hyperglycemia, reduced insulin sensitivity, hypertriglyceridemia, and hypertension, all of which increase CV risk (36, 37, 54). In line with these observations, we found an increase in CV risk in our study, in the opposite direction to its trend in the early stages of the multidisciplinary program. Perrone et al. showed that the reduction of daily physical activity during the lockdown period in dyslipidemic patients resulted in an increase in LDL-cholesterol levels of 15.8%, leading to an increased CV risk (55); this had already been shown by Sung et al. in healthy athletes following the interruption of physical activity for 1 month (56) and is also reflected in our results, which document an increase in total cholesterol levels after an initial improvement in the first year of intervention. Often, in addition to the worsening of laboratory parameters, an increase in body weight during lockdown has been documented, not infrequently linked to an increased caloric intake, which was not matched by increased physical activity, or to the worsening of diet quality (31, 57–59). In addition, home confinement has often also resulted in increased alcohol and tobacco consumption (33, 38, 60–62), as also highlighted in our study; on the contrary, indirectly, the pandemic has led to a worsening of hypertension, mainly due to reduced access to doctors' surgeries and pharmacies for the supply of drugs (32, 34, 35, 63); this aspect, on the other hand, does not seem to be correlated with confinement *per se*, as demonstrated by Pengo et al. who did not detect major changes in blood pressure levels during lockdown by means of a telemonitoring system for patients (64, 65); Similarly, in our study, SBP was the only CV risk factor that continued to improve during the pandemic phase.

The stratification of our results according to the presence of diabetes and/or hypertension and physical activity/inactivity did not issue any significant results, and this can be due to the limited number of people in different subgroups. Nonetheless, the relative reduction of CRS was higher in people with diabetes and/or hypertension and physically inactive patients but these results should be considered exploratory (8, 66). However, literature shows that the implementation of evidence-based guidelines in daily clinical practice may encounter difficulties, resulting in poor outcomes and less impact on CVD risk (67). This is reported by Kotseva et al. (67), who observed insufficient control of CVD risk factors such as blood pressure, cholesterol, and diabetes among patients followed over time in the EUROASPIRE studies, albeit reporting slightly more encouraging results than in the European EURIKA study (68). One of the aspects that can be highlighted in the EUROASPIRE studies is the absence of multidisciplinary follow-up by healthcare professionals: this may explain how our study, albeit on a considerably smaller sample, may have achieved better blood pressure control and an initial reduction in CVD risk, which then stopped when follow-up ceased.

In our analysis, we focused on individuals presenting at the GP's practice. The role of GPs is very important in patient care and is useful for risk assessment and communication at the individual level in order to guide lifestyle modification and treatment decisions (69, 70). Furthermore, primary healthcare and community nurses play a significant role in improving patients' self-management (71). Htay et al. (29), in a systematic review, highlighted the importance of the advanced nurse practitioner (ANP) in improving health outcomes and patient satisfaction in different care settings, including primary care. Therefore, the ANP plays the role of facilitator and, together with the physician, has a synergistic action in reducing mortality given by CVDs (30, 72). Community-based system interventions represent a promising but complex approach to preventive strategies. Existing studies suggest that the implementation of multiple actions by engaged community leaders (steering committees) is of critical importance to influence a complex system.

This study has several limitations. First, the main weakness of this study lies in the potential “self-selection bias,” as participation implied a willingness of the respondent to take an active part in the study and this willingness could be influenced by certain characteristics that could act as confounding factors. Furthermore, since the study has not foreseen a control group, observed changes should be commented on with caution as they could have also been attributable to factors other than the intervention. Moreover, not all patients included in the study were included in the analysis, due to missing data during the follow-up at 6, 12, and 18 months. Furthermore, our sample size was relatively small and no sample size determination or power analysis has been performed because it was a pilot study. Eventually, the generalizability of our findings is limited to people with a moderate risk profile. Furthermore, it should be observed that the pandemic outbreak and the following national lockdown (from March to May 2020) have probably affected results at 18 months; however, it is difficult to estimate their real influence and link them to changes in lifestyle and adherence to healthy behaviors.

Finally, although we documented at baseline the number of subjects on antihypertensive and cholesterolaemic-lowering therapy, we did not acquire information on the type of drugs nor on the dosage used: we are, therefore, unable to assess the impact of the program on the therapy of the patients involved by evaluating whether the dosage changed during the course of the study.

The results of this study corroborate the positive impact of health promotion and prevention interventions; however, randomized control trials are needed to further investigate the effectiveness of such kinds of interventions. This could guarantee actively taking charge of subjects by identifying potentially problematic situations and implementing policies for the prevention of risk factors of CVD and other non-communicable diseases.

## Data availability statement

The datasets presented in this article are not readily available because only GPs hold patients' clinical data and they are responsible for them. Data have been anonymized and transmitted to the Section of Hygiene of Università Cattolica del Sacro Cuore, and they are available on request from the authors. Requests to access the datasets should be directed to PL, patrizia.laurenti@unicatt.it.

## Ethics statement

The studies involving human participants were reviewed and approved by Ethical Committee Lazio 1 c/o San Camillo Forlanini Hospital (with protocol number 1817 / CE Lazio 1). The patients/participants provided their written informed consent to participate in this study.

## Author contributions

PL, GD, AT, and SG: conceptualization and project administration. CG, EMaz, AL, EMar, and FD'A: investigation. CW, PL, and GD: methodology. AL, EMar, and FD'A: writing–original draft. AT, CG, PL, VG, AM, MP, and AB: writing–review and editing. CW: software and formal analysis. AT, CG, EMaz, and SG: data curation. PL and CW: validation. AT, CG, PL, VG, AM, MP, AB, and GD: supervision. All authors have read and agreed to the published version of the manuscript.

## References

[1] World Health Organization. Cardiovascular Diseases (CVDs). Available online at: https://www.who.int/news-room/fact-sheets/detail/cardiovascular-diseases-(cvds) (accessed October 12, 2022).

[2] Heart Disease and Stroke Statistics-2021 Update A Report from the American Heart Association. Circulation. (2021) 134:E254–E743. 10.1161/CIR.0000000000000950 33501848 PMC13036842

[3] Wilkins E Wilson L Wickramasinghe K Bhatnagar P Leal J Luengo-Fernandez R. European Cardiovascular Disease Statistics 2017. Brussels: European Heart Network (2017).

[4] ISS. Il Progetto Cuore. Available online at: https://www.cuore.iss.it/altro/cuore (accessed December 31, 2021).

[5] Pyörälä K. CHD prevention in clinical practice. Lancet. (1996) 348 (Suppl 1). 10.1016/S0140-6736(96)98009-5 8918526

[6] Perk J de Backer G Gohlke H Graham I Reiner Ž Verschuren M. European Guidelines on cardiovascular disease prevention in clinical practice (version 2012). The Fifth Joint Task Force of the European Society of Cardiology and Other Societies on Cardiovascular Disease Prevention in Clinical Practice (constituted by representatives of nine societies and by invited experts). Eur Heart J. (2012) 33:1635–701. 10.1093/eurheartj/ehs092 22555213

[7] Sisti LG Dajko M Campanella P Shkurti E Ricciardi W de Waure C. The effect of multifactorial lifestyle interventions on cardiovascular risk factors: a systematic review and meta-analysis of trials conducted in the general population and high risk groups. Prev Med (Baltim). (2018) 109:82–97. 10.1016/j.ypmed.2017.12.027 29291422

[8] Rippe JM. Lifestyle Strategies for Risk Factor Reduction, Prevention, and Treatment of Cardiovascular Disease. Am J Lifestyle Med. (2019) 13:204. 10.1177/1559827618812395 30800027 PMC6378495

[9] Harrison C Britt H Miller G Henderson J. Prevalence of Chronic Conditions in Australia. PLoS ONE. (2013) 8:e67494. 10.1371/journal.pone.0067494 23935834 PMC3720806

[10] Califf RM Armstrong PW Carver JR D'Agostino RB Strauss WE. 27th Bethesda Conference: matching the intensity of risk factor management with the hazard for coronary disease events. Task Force 5 Stratification of patients into high, medium and low risk subgroups for purposes of risk factor management. J Am Coll Cardiol. (1996) 27:1007–19. 10.1016/0735-1097(96)87733-3 8609316

[11] Greenland P Alpert JS Beller GA Benjamin EJ Budoff MJ Fayad ZA. 2010 ACCF/AHA guideline for assessment of cardiovascular risk in asymptomatic adults: a report of the American College of Cardiology Foundation/American Heart Association Task Force on Practice Guidelines. Circulation. (2010) 122. 10.1161/CIR.0b013e3182051b4c 21098428

[12] Conroy RM Pyörälä K Fitzgerald AP Sans S Menotti A De Backer G. Estimation of ten-year risk of fatal cardiovascular disease in Europe: the SCORE project. Eur Heart J. (2003) 24:987–1003. 10.1016/S0195-668X(03)00114-3 12788299

[13] Lee MS Flammer AJ Li J Lennon RJ Delacroix S Kim H. Comparison of Time trends of cardiovascular disease risk factors and framingham risk score between patients with and without acute coronary syndrome undergoing percutaneous intervention over the last 17 years: from the mayo clinic percutaneous coronary intervention registry. Clin Cardiol. (2015) 38:747. 10.1002/clc.22484 26671071 PMC6490742

[14] ASCVD Risk Estimator. Available online at: https://tools.acc.org/ldl/ascvd_risk_estimator/index.html#!/calulate/estimator/patient (accessed October 12, 2022).

[15] Jancey J James A Lee A Howat P Hills AP Anderson AS. Metabolic syndrome in rural Australia: An opportunity for primary health care. Aust J Rural Health. (2019) 27:210–5. 10.1111/ajr.12598 31062903

[16] D'Agostino RB Grundy S Sullivan LM Wilson P. Validation of the Framingham coronary heart disease prediction scores: results of a multiple ethnic groups investigation. JAMA. (2001) 286:180–7. 10.1001/jama.286.2.180 11448281

[17] Anderson KM Wilson PWF Odell PM Kannel WB. An updated coronary risk profile. A statement for health professionals. Circulation. (1991) 83:356–62. 10.1161/01.CIR.83.1.356 1984895

[18] Wilson PWF D'Agostino RB Levy D Belanger AM Silbershatz H Kannel WB. Prediction of coronary heart disease using risk factor categories. Circulation. (1998) 97:1837–47. 10.1161/01.CIR.97.18.1837 9603539

[19] de Backer G Ambrosioni E Borch-Johnsen K Brotons C Cifkova R Dallongeville J. European guidelines on cardiovascular disease prevention in clinical practice. Third joint task force of european and other societies on cardiovascular disease prevention in clinical practice. Eur Heart J. (2003) 24:1601–10. 10.1016/S0195-668X(03)00347-6 12964575

[20] Haskell WL Alderman EL Fair JM Maron DJ Mackey SF Superko HR. Effects of intensive multiple risk factor reduction on coronary atherosclerosis and clinical cardiac events in men and women with coronary artery disease. The Stanford Coronary Risk Intervention Project (SCRIP). Circulation. (1994) 89:975–90. 10.1161/01.CIR.89.3.975 8124838

[21] Saggese L Scala E. Comune di Roma. Il Contesto territoriale e il profilo socio-demografico del territorio e della comunità locale. (2015). Available online at: https://www.comune.roma.it/PCR/resources/cms/documents/cap_1_contesto_territoriale_profilo_socio_demografico.pdf (accessed April 20, 2022).

[22] Whelton PK Carey RM Aronow WS Casey DE Collins KJ Himmelfarb CD. 2017 ACC/AHA/AAPA/ABC/ACPM/AGS/APhA/ ASH/ASPC/NMA/PCNA guideline for the prevention, detection, evaluation, and management of high blood pressure in adults a report of the American College of Cardiology/American Heart Association Task Force on Clinical practice. Hypertension. (2018) 71: 13–115. 10.1161/HYP.0000000000000065 29133356

[23] Drinking Levels Defined | National Institute on Alcohol Abuse and Alcoholism (NIAAA). Available online at: https://www.niaaa.nih.gov/alcohol-health/overview-alcohol-consumption/moderate-binge-drinking (accessed April 20, 2022).

[24] Istituto Superiore di Sanità. Il Progetto CUORE. Available online at: http://progettocuore.medisoft.it/cuore_exe/registration.aspx (accessed October 14, 2022).

[25] Sorveglianza Passi. Available online at: https://www.epicentro.iss.it/passi/infoPassi/infoGen (accessed October 14, 2022).

[26] Wallston BS Wallston KA Kaplan GD Maides SA. Development and validation of the health locus of control (HLC) scale. J Consult Clin Psychol. (1976) 44:580–5. 10.1037/0022-006X.44.4.580 939841

[27] Zimet GD Dahlem NW Zimet SG Farley GK. The Multidimensional Scale of Perceived Social Support. J Pers Assess. (1988) 52:30–41. 10.1207/s15327752jpa5201_2

[28] Prochaska JO di Clemente CC. Transtheoretical therapy: Toward a more integrative model of change. Psychotherapy. (1982) 19:276–88. 10.1037/h0088437

[29] Htay M Whitehead D. The effectiveness of the role of advanced nurse practitioners compared to physician-led or usual care: a systematic review. Int J Nurs Stud Adv. (2021) 3:100034. 10.1016/j.ijnsa.2021.100034 PMC11080477 38746729

[30] Connolly SB Kotseva K Jennings C Atrey A Jones J Brown A. Outcomes of an integrated community-based nurse-led cardiovascular disease prevention programme. Heart. (2017) 103:840–7. 10.1136/heartjnl-2016-310477 28255098 PMC5566096

[31] Özden G Parlar Kiliç S. The effect of social isolation during COVID-19 pandemic on nutrition and exercise behaviors of nursing students. Ecol Food Nutr. (2021) 60:663–81. 10.1080/03670244.2021.1875456 33475005

[32] Burnier M. Controversies in the management of patients with arterial hypertension. Kardiol Polska. (2019) 77:902–7. 10.33963/KP.15002 31571674

[33] Dyal SR Valente TW. A systematic review of loneliness and smoking: Small effects, big implications. Subst Use Misuse. (2015) 50:1697–716. 10.3109/10826084.2015.1027933 26555089 PMC4803029

[34] Weber T. The corona-virus disease 2019 pandemic compromised routine care for hypertension: a survey conducted among excellence centers of the European Society of Hypertension. J Hypertens. (2021) 39:190–5. 10.1097/HJH.0000000000002703 33273364

[35] Kauppi M Elovainio M Stenholm S Virtanen M Aalto V Koskenvuo M. Social networks and patterns of health risk behaviours over two decades: a multi-cohort study. J Psychosom Res. (2017) 99:45–58. 10.1016/j.jpsychores.2017.06.003 28712430

[36] Gupta N Hallman DM Mathiassen SE Aadahl M Jørgensen MB Holtermann A. Are temporal patterns of sitting associated with obesity among blue-collar workers? A cross sectional study using accelerometers. BMC Public Health. (2016) 16:1–10. 10.1186/s12889-016-2803-9 26872944 PMC4752751

[37] Betts JA Smith HA Johnson-Bonson DA Ellis TI Dagnall J Hengist A. The energy cost of sitting versus standing naturally in man. Med Sci Sports Exerc. (2019) 51:726–33. 10.1249/MSS.0000000000001841 30673688

[38] Bérard E Huo Yung Kai S Coley N Bongard V Ferrières J. One-Year Impact of COVID-19 Lockdown-Related Factors on Cardiovascular Risk and Mental Health: A Population-Based Cohort Study. Int J Environ Res Public Health. (2022) 19. 10.3390/ijerph19031684 35162707 PMC8835147

[39] Arnett DK Blumenthal RS Albert MA Buroker AB Goldberger ZD Hahn EJ. 2019 ACC/AHA guideline on the primary prevention of cardiovascular disease: a report of the American College of Cardiology/American Heart Association Task Force on Clinical Practice Guidelines. Circulation. (2019) 140:e596–646. 10.1161/CIR.0000000000000678 30879355 PMC7734661

[40] 2018 Physical Activity Guidelines Advisory Committee. 2018 Physical Activity Guidelines Advisory Committee Scientific Report. Washington, DC: U.S. Department of Health and Human Services (2018).

[41] Cleeman JI. Executive Summary of The Third Report of The National Cholesterol Education Program (NCEP) Expert Panel on Detection, Evaluation, And Treatment of High Blood Cholesterol In Adults (Adult Treatment Panel III). JAMA. (2001) 285:2486–97. 10.1001/jama.285.19.2486 11368702

[42] American College of Lifestyle Medicine. Available online at: https://www.lifestylemedicine.org/ (accessed October 12, 2022).

[43] Yusuf S Wood D Ralston J Reddy KS. The World Heart Federation's vision for worldwide cardiovascular disease prevention. Lancet. (2015) 386:399–402. 10.1016/S0140-6736(15)60265-3 25892680

[44] Stewart J Addy K Campbell S Wilkinson P. Primary prevention of cardiovascular disease: Updated review of contemporary guidance and literature. JRSM Cardiovasc Dis. (2020) 9:204800402094932. 10.1177/2048004020949326 32994926 PMC7502686

[45] Jennings C Astin F. A multidisciplinary approach to prevention. Eur J Prev Cardiol. (2017) 24:77–87. 10.1177/2047487317709118 28618913

[46] Jain A Davis AM. Primary prevention of cardiovascular disease. JAMA. (2019) 322:1817–8. 10.1001/jama.2019.15915 31584604

[47] Pennant M Davenport C Bayliss S Greenheld W Marshall T Hyde C. Community programs for the prevention of cardiovascular disease: a systematic review. Am J Epidemiol. (2010) 172:501–16. 10.1093/aje/kwq171 20667932

[48] Patnode CD Evans CV Senger CA Redmond N Lin JS. Behavioral Counseling to Promote a Healthful Diet and Physical Activity for Cardiovascular Disease Prevention in Adults Without Known Cardiovascular Disease Risk Factors: Updated Systematic Review for the U.S. Preventive Services Task Force. HRQ Publication No 15-05222-EF-1. Rockville, MD: Agency for Healthcare Research and Quality, JAMA. (2017). 10.1001/jama.2017.3303 29364620

[49] Lewington S Clarke R Qizilbash N Peto R Collins R. Age-specific relevance of usual blood pressure to vascular mortality: a meta-analysis of individual data for one million adults in 61 prospective studies. Lancet. (2002) 360:1903–13. 10.1016/S0140-6736(02)11911-8 12493255

[50] Lönnberg L Ekblom-Bak E Damberg M. Reduced 10-year risk of developing cardiovascular disease after participating in a lifestyle programme in primary care. Ups J Med Sci. (2020) 125:250–6. 10.1080/03009734.2020.1726533 32077778 PMC7720946

[51] Silagy C Stead L. Physician advice for smoking cessation. Cochrane Database of Syst Rev. (2001). 10.1002/14651858.CD000165 11405953

[52] Coronavirus, le misure adottate dal Governo | www.governo.it. Available online at: https://www.governo.it/it/coronavirus-misure-del-governo (accessed October 4, 2022).

[53] Chandrasekaran B Ganesan TB. Sedentarism and chronic disease risk in COVID 19 lockdown - a scoping review. Scott Med J. (2021) 66:3–10. 10.1177/0036933020946336 32718266 PMC8685753

[54] Saunders TJ Atkinson HF Burr J MacEwen B Skeaff CM Peddie MC. The acute metabolic and vascular impact of interrupting prolonged sitting: a systematic review and meta-analysis. Sports Med. (2018) 48:2347–66. 10.1007/s40279-018-0963-8 30078066

[55] Perrone MA Feola A Pieri M Donatucci B Salimei C Lombardo M. The Effects of Reduced Physical Activity on the Lipid Profile in Patients with High Cardiovascular Risk during COVID-19 Lockdown. Int J Environ Res Public Health. (2021) 18. 10.3390/ijerph18168858 34444607 PMC8393411

[56] Sung YC Liao YH Chen CY Chen YL Chou CC. Acute changes in blood lipid profiles and metabolic risk factors in collegiate elite taekwondo athletes after short-term de-training: a prospective insight for athletic health management. Lipids Health Dis. (2017) 16. 10.1186/s12944-017-0534-2 28738856 PMC5525308

[57] Freiberg A Schubert M Starke KR Hegewald J Seidler A. A rapid review on the influence of COVID-19 lockdown and quarantine measures on modifiable cardiovascular risk factors in the general population. Int J Environ Res Public Health. (2021) 18. 10.3390/ijerph18168567 34444316 PMC8393482

[58] Unubol M Erdemir Z Colak G Guney E Omurlu IK. Obesity pandemic triggered by the COVID-19 pandemic: experience from Turkey. Pol Arch Intern Med. (2021) 131:766–8. 10.20452/pamw.16037 34165269

[59] Kreutz R Dobrowolski P Prejbisz A Algharably EAEH Bilo G Creutzig F. Lifestyle, psychological, socioeconomic and environmental factors and their impact on hypertension during the coronavirus disease 2019 pandemic. J Hypertens. (2021) 39:1077–89. 10.1097/HJH.0000000000002770 33395152

[60] Kelly S Olanrewaju O Cowan A Brayne C Lafortune L. Alcohol and older people: A systematic review of barriers, facilitators and context of drinking in older people and implications for intervention design. PLoS ONE. (2018) 13. 10.1371/journal.pone.0191189 29370214 PMC5784942

[61] Stanesby O Labhart F Dietze P Wright CJC Kuntsche E. The contexts of heavy drinking: A systematic review of the combinations of context-related factors associated with heavy drinking occasions. PLoS ONE. (2019) 14. 10.1371/journal.pone.0218465 31291261 PMC6619678

[62] Seo DC Huang Y. Systematic review of social network analysis in adolescent cigarette smoking behavior^*^. J Sch Health. (2012) 82:21–7. 10.1111/j.1746-1561.2011.00663.x 22142171

[63] Cuffee Y Ogedegbe C Williams NJ Ogedegbe G Schoenthaler A. Psychosocial risk factors for hypertension: an update of the literature. Curr Hypertens Rep. (2014) 16. 10.1007/s11906-014-0483-3 25139781 PMC4163921

[64] Pengo M Albini F Guglielmi G Mollica C Soranna D Zambra G. Home blood pressure during COVID-19 related lockdown in patients with hypertension. Eur J Prev Cardiol. (2021) 28. 10.1093/eurjpc/zwab061.142 33899916 PMC8135487

[65] Januszewicz A Wojciechowska W Prejbisz A Dobrowolski P Rajzer M Kreutz R. Impact of the COVID-19 pandemic on blood pressure control and cardiovascular risk profile in patients with hypertension. Pol Arch Intern Med. (2021) 131. 10.20452/pamw.16129 34704702

[66] Moore SC Patel AV Matthews CE Berrington de Gonzalez A Park Y Katki HA. Leisure time physical activity of moderate to vigorous intensity and mortality: a large pooled cohort analysis. PLoS Med. (2012) 9. 10.1371/journal.pmed.1001335 23139642 PMC3491006

[67] Kotseva K de Backer G de Bacquer D Ryden L Hoes A Grobbee D. Primary prevention efforts are poorly developed in people at high cardiovascular risk: a report from the European Society of Cardiology EURObservational Research Programme EUROASPIRE V survey in 16 European countries. Eur J Prev Cardiol. (2021) 28:370–9. 10.1177/2047487320908698 33966079

[68] Banegas JR López-García E Dallongeville J Guallar E Halcox JP Borghi C. Achievement of treatment goals for primary prevention of cardiovascular disease in clinical practice across Europe: the EURIKA study. Eur Heart J. (2011) 32:2143–52. 10.1093/eurheartj/ehr080 21471134 PMC3164103

[69] Beganlic A Pavljasevic S Kreitmayer S Zildzic M Softic A Selmanovic S. Qualitative evaluation of cardiovascular diseases management in family medicine team in one year level. Med Arch. (2015) 69:140. 10.5455/medarh.2015.69.140-144 26261378 PMC4500297

[70] Diederichs C Neuhauser H Rücker V Busch MA Keil U Fitzgerald AP. Predicted 10-year risk of cardiovascular mortality in the 40 to 69 year old general population without cardiovascular diseases in Germany. PLoS ONE. (2018) 13. 10.1371/journal.pone.0190441 29293619 PMC5749805

[71] Simonetti V Comparcini D Tomietto M Pavone D Flacco ME Cicolini G. Effectiveness of a family nurse-led programme on accuracy of blood pressure self-measurement: a randomised controlled trial. J Clin Nurs. (2021) 30:2409–19. 10.1111/jocn.15784 33872417

[72] Kuneinen SM Eriksson JG Kautiainen H Ekblad MO Korhonen PE. The feasibility and outcome of a community-based primary prevention program for cardiovascular disease in the 21st century. Scand J Prim Health Care. (2021) 39:157–65. 10.1080/02813432.2021.1913893 34092186 PMC8293959

